# Digital Step Therapy: A Smart Framework for Payer Adoption of Prescription Digital Therapeutics

**DOI:** 10.7759/cureus.84079

**Published:** 2025-05-14

**Authors:** Shaheen E Lakhan

**Affiliations:** 1 Medical Office, Click Therapeutics, Inc., New York City, USA; 2 Neurology, Western University of Health Sciences, Pomona, USA; 3 Neurology, A.T. Still University School of Osteopathic Medicine in Arizona, Mesa, USA; 4 Neurology, Morehouse School of Medicine, Atlanta, USA; 5 Bioscience, Global Neuroscience Initiative Foundation, Miami, USA

**Keywords:** clinical pathways, ct-132, digital step therapy, fda authorization, health equity, migraine prevention, patient-reported outcomes, prescription digital therapeutics, real-world evidence, value-based care

## Abstract

As prescription digital therapeutics (PDTs) gain FDA authorization and clinical traction, health insurers and policymakers face a growing imperative to define reimbursement strategies that ensure equitable access while delivering demonstrable value. We propose digital step therapy: a modernized framework in which patients initiate care with evidence-based PDTs before escalating to more intensive or costly interventions. Unlike traditional step therapy, which often enforces rigid fail-first hierarchies, digital step therapy can leverage real-time engagement data, patient-reported outcomes (PROs), and modular design features unique to software-based therapeutics.

This editorial outlines the rationale, opportunities, risks, and implementation principles for a smart, patient-centered digital step therapy model. It emphasizes the need for clinician override mechanisms, equity-driven usability standards, and regulatory alignment to avoid replicating historical barriers to care. Using CT-132 (Click Therapeutics, Inc., New York, NY, USA) for episodic migraine as an illustrative case, we demonstrate how digital step therapy can deliver a clinically adaptive, data-informed, and payer-aligned approach that promotes early access to safe, scalable interventions, acknowledging implementation barriers, while continuously refining care pathways based on real-world outcomes.

## Editorial

Prescription digital therapeutics (PDTs) have emerged as a new class of FDA-authorized, evidence-based software interventions delivered via smartphone or tablet, designed to treat a wide range of medical and psychiatric conditions, including major depressive disorder, attention-deficit/hyperactivity disorder, migraine, irritable bowel syndrome, and more [1]. These digital interventions are held to rigorous regulatory standards for safety, efficacy, and usability, distinguishing them from unregulated wellness apps and marking a significant evolution in therapeutic modalities.

As clinical evidence supporting PDTs continues to grow and their market presence expands, health insurers and pharmacy benefit managers (PBMs) face urgent questions about how to equitably and efficiently integrate these therapies into reimbursement and care delivery systems. Traditional step therapy protocols, often used to control pharmaceutical costs by requiring failure on lower-cost treatments before advancing to more expensive options, may offer a starting blueprint. Yet applying these models to PDTs without adaptation risks misalignment with digital therapeutic functionality, patient engagement dynamics, and real-world effectiveness data.

This editorial introduces and advocates for a next-generation framework: digital step therapy. Unlike its pharmacologic predecessor, digital step therapy is built around the unique strengths of PDTs, real-time data capture, modular design, low systemic risk, and patient-level insights to support adaptive care pathways that are both value-based and patient-centered.

We argue that, when thoughtfully designed, digital step therapy can optimize resource allocation, promote innovation, and improve clinical outcomes. However, without appropriate safeguards, such as clinician override mechanisms, real-world evidence generation, and equity-focused design, it could reproduce the rigid gatekeeping of traditional models and restrict access for vulnerable populations.

This paper outlines the rationale, opportunities, risks, and design principles for digital step therapy, including an illustrative example for migraine management, offering a forward-looking framework that reflects the evolving digital landscape of therapeutic care.

Opportunities for digital step therapy

When thoughtfully designed and equitably implemented, digital step therapy holds transformative potential for both clinical outcomes and healthcare system efficiency. It offers a future-forward model of evidence-based sequencing, in which patients initiate care with PDTs that have demonstrated safety, efficacy, and scalability before progressing to more intensive, costly, or invasive interventions. For example, a PDT delivering cognitive behavioral therapy for insomnia [2] or sensory, autonomic, and affective neuromodulation for migraine [3] may serve as an effective first-line option, reducing reliance on sedating and cognitive-impairing classes of medication. This approach can minimize unnecessary polypharmacy, reduce the risk of side effects and drug interactions, and empower patients to engage more actively in their own care.

Unlike traditional pharmacologic agents, PDTs are uniquely suited to generate continuous, real-world data. Engagement metrics, adherence rates, symptom trajectories, and patient-reported outcomes (PROs) can be tracked in real time, creating dynamic feedback loops between patients, providers, and payers. This data infrastructure enables adaptive care pathways, earlier detection of non-response, and fine-tuning of step progression criteria based on lived clinical experience. 

Additionally, a well-calibrated digital step therapy framework can incentivize innovation. By clearly linking coverage decisions to real-world performance, such frameworks reward PDT developers who invest in usability testing, population-specific validation, and health equity by design. This can accelerate market access, outcomes-based pricing negotiations, and trust among stakeholders.

Ultimately, digital step therapy can catalyze a more learning-oriented, patient-centered health system, one that adapts in real time, aligns with modern regulatory expectations, and delivers the right intervention at the right time through the right modality.

Risks of overly rigid implementation

While digital step therapy offers the promise of structured, evidence-based care pathways, its benefits can be quickly undermined if implemented in a rigid or algorithmic manner that fails to reflect the complexity of clinical practice. One major risk is the replication of “fail-first” dynamics seen in traditional step therapy, where patients must demonstrate failure on lower-tier options before accessing more appropriate treatments. Overly prescriptive hierarchies that lack clinician override mechanisms can delay effective care, frustrate providers, and erode patient trust.

This concern is particularly acute in behavioral health and neurologic conditions, where therapeutic fit and patient preference significantly influence adherence and outcomes. Forcing patients to endure ill-suited digital tools, especially when engagement and personalization are key determinants of efficacy, may lead to premature dropout, symptom worsening, or disengagement from care entirely.

Compounding the challenge is the limited comparative effectiveness evidence across PDTs. With few head-to-head trials and sparse long-term real-world data, sequencing decisions may be based more on theoretical models or payer preference than proven clinical superiority. This undermines both the scientific and ethical foundation of digital step therapy.

Finally, health equity risks loom large. If early-step PDTs are not designed with inclusivity in mind, taking into account language, literacy, culture, broadband access, and disability status, they may inadvertently amplify disparities among historically underserved populations. Without careful attention to accessibility and representation in validation studies, rigid protocols may systematically exclude the very patients who stand to benefit most from digitally enabled care.

In short, without built-in flexibility, transparency, and safeguards, digital step therapy can entrench the very barriers it seeks to dismantle.

Additional barriers include payer concerns around pricing, cost-effectiveness relative to generics, and the operational burden of protocol integration. Without streamlined digital formularies and value-aligned incentives, adoption may be uneven or delayed. Addressing these concerns through real-world data and outcomes-based contracts will be key to broader uptake.

Design principles for a smart framework

To balance standardization with flexibility, digital step therapy protocols must be built on robust, multi-stakeholder design features (Table 1). This includes clear clinical logic, transparency, real-world validation, and equity by design. Protocols should preserve clinician autonomy through override mechanisms that support professional judgment and exceptions for atypical cases. They must integrate real-world data, including engagement, adherence, and clinical effectiveness, to refine and personalize step progression. PROs, such as the Patient Global Impression of Change (PGI-C) and quality of life, should be tracked to ensure meaningful benefit. Inclusivity is critical; early-step PDTs must be linguistically and culturally adaptive, accessible across device types, and validated in diverse populations. Stakeholder co-design is essential; payers, regulators, clinicians, patients, and developers should collaborate on step protocols, coverage criteria, and evaluation metrics. Usability should be independently validated, including testing for interface clarity, accessibility, and retention performance. Finally, protocols must align with regulatory standards, including FDA labeling, Health Insurance Portability and Accountability Act (HIPAA)-compliant data practices, and HL7 Fast Healthcare Interoperability Resources (FHIR) interoperability frameworks [4].

**Table 1:** Design checklist for digital step therapy protocols This author-created table outlines the foundational domains, principles, and operational criteria required to ensure that digital step therapy pathways are clinically sound, patient-centered, technologically interoperable, and policy-compliant. It serves as a practical guide for payers, regulators, and developers to co-create protocols that leverage the unique strengths of prescription digital therapeutics (PDTs) while safeguarding against unintended barriers to access and equity.

Domain	Design principle	Operational criteria
Clinical	Clinician override mechanisms	Step exceptions documented in electronic health records (EHR) with payer-facing override codes
Outcomes	Real-world performance	Data from EHR and in-app patient-reported outcomes reviewed periodically for protocol refinement
Usability	Validated patient engagement and accessibility	Multilingual user interface (UI), minimal dropout in first weeks, maximal task/module/lesson adherence
Equity	Inclusive design	Accessibility for low-bandwidth, underserved, and disability populations
Governance	Stakeholder co-design	Advisory panel includes clinicians, people with lived experience, and payers
Interoperability	Standards-based data integration	HL7 Fast Healthcare Interoperability Resources (FHIR) compliance for automated coverage, tracking, and quality monitoring
Regulatory Alignment	FDA-label conformity and safety transparency	Use restricted to cleared indications; safety summary incorporated in protocol

Policy recommendations

To operationalize digital step therapy at scale, a coordinated policy blueprint is needed, spanning federal agencies, state programs, and private-sector payers. The Centers for Medicare & Medicaid Services (CMS) Innovation Center should spearhead pilot programs that test digital-first care models for high-burden, high-cost conditions such as migraine, depression, and schizophrenia, where evidence-based digital therapeutics are emerging. These pilots should be embedded within Medicaid and Medicare populations to evaluate real-world feasibility and outcomes across diverse, often underserved, groups. These pilots could test not only clinical outcomes but also patient-reported outcomes, engagement metrics, and health equity indicators.

Commercial payers should implement coverage with evidence development (CED) models to provisionally reimburse PDTs while gathering real-world effectiveness and utilization data. These arrangements de-risk early adoption and provide a clear, data-driven pathway from limited to full coverage status. A strong international precedent is Germany’s Fast-Track Process for Digitale Gesundheitsanwendungen (DiGA), which allows digital health applications to be temporarily listed for reimbursement under statutory health insurance while real-world evidence is collected [5]. DiGA has demonstrated how a centralized, regulatory-backed mechanism can facilitate both innovation and accountability, offering conditional access with time-bound expectations for data generation and outcomes reporting. U.S. commercial payers can adapt a similar model by reimbursing PDTs with provisional status contingent on post-market performance metrics, such as engagement, clinical outcomes, and healthcare utilization impact, thereby balancing early access with ongoing evaluation. Embedding this model within digital step therapy protocols can help establish a learning healthcare system that continuously refines coverage decisions based on emerging evidence. These strategies also address common payer concerns around high upfront costs and the relative value of PDTs compared to generic pharmacotherapies. By tying reimbursement to clinical outcomes, rather than static pricing, payers can ensure that digital therapeutics deliver measurable value before scaling access.

PBMs and health plans should harmonize digital formularies by establishing standardized criteria for usability, clinical performance, and patient engagement. These criteria should include validated metrics such as task adherence, dropout rates, and user experience benchmarks to ensure PDTs meet a consistent threshold of therapeutic quality. Outcomes-based contracts can further align incentives, tying reimbursement to real-world effectiveness rather than static pricing models. By embedding PDTs into value-based payment arrangements, PBMs and payers can unlock shared savings when digital step therapy reduces avoidable costs, such as hospitalizations, medication overuse, or specialist visits. This shift from volume-based to value-based reimbursement aligns with broader healthcare reform trends and can catalyze a more accountable, evidence-driven digital therapeutic ecosystem. Moreover, transparent coverage protocols, including clear criteria for initiation, continuation, and escalation, help providers and patients navigate digital formularies with confidence and consistency. Collectively, these measures will accelerate the integration of PDTs into mainstream care while preserving flexibility for innovation and individualized treatment pathways.

Standards bodies like HL7 should expedite the development and universal adoption of interoperability protocols that enable seamless integration of PDT data into clinical and administrative systems [4]. This includes structured data capture for engagement metrics, adherence trends, clinical outcomes, and patient-reported measures such as PGI-C and MIDAS. Interoperability with electronic health records (EHRs), payer claims systems, and quality registries ensures that PDTs are not siloed apps but embedded components of care delivery and evaluation. Real-time data exchange allows clinicians to monitor patient progress and make timely adjustments, supports payers in validating value-based contracts, and empowers regulators to assess safety and performance post-market. Adoption of HL7 FHIR standards is critical to achieving this vision, providing a common framework for secure, scalable, and privacy-compliant data flow [4]. Without this infrastructure, the full potential of digital step therapy, including adaptive protocols, predictive analytics, and continuous learning health systems, cannot be realized.

Finally, researchers, digital therapeutic developers, and funders must prioritize a new generation of rigorous evidence generation tailored to the unique nature of PDTs. This includes comparative effectiveness research, where multiple PDTs or PDTs versus standard of care are evaluated for clinical and economic outcomes in diverse populations. Head-to-head trials are especially critical, given the proliferation of PDTs targeting overlapping conditions with varying levels of validation and usability. Without direct comparisons, payers and clinicians lack the data needed to inform optimal sequencing and formulary inclusion.

Equally important is pragmatic implementation research, studies embedded within real-world care settings that assess not only clinical efficacy but also workflow integration, provider and patient adoption, adherence, and equity. These investigations should leverage hybrid trial designs, digital biomarkers, and federated data platforms to accelerate insights while minimizing burden.

This evidence will be indispensable for refining digital step therapy protocols, supporting value-based reimbursement decisions, and sustaining innovation. Public and private funders, including the National Institutes of Health, Patient-Centered Outcomes Research Institute, Biomedical Advanced Research and Development Authority, and venture partners, should consider dedicated initiatives for digital therapeutics evaluation, with an emphasis on replicability, diversity, and health system impact. In short, robust evidence generation is not just a regulatory or academic exercise; it is a strategic imperative for long-term viability and trust in this new frontier of medicine.

Illustrative case study of digital step therapy for migraine

Digital step therapy is most effective when grounded in both clinical precedent and evolving standards of care. While innovations in PDTs and the newer class of calcitonin gene-related-peptide (CGRP)-targeting therapies have rapidly advanced migraine management since their first marketing in 2018, formal clinical guidelines have lagged behind. The American Academy of Neurology (AAN), for instance, has not updated its migraine prevention guidelines since 2012 [6], leaving a significant decade-long gap in consensus-based recommendations. In this vacuum, recent position statements such as the 2024 American Headache Society (AHS) update play an increasingly pivotal role in informing clinical decisions and payer policy [7]. AHS updated its position to state that CGRP inhibitors should be considered first-line options for the prevention of migraine, without requiring failure of non-specific preventive medications. This shift acknowledges the robust clinical trial and real-world evidence supporting the efficacy, safety, and tolerability of this drug class.

In April 2025, the FDA authorized CT-132 (Click Therapeutics, Inc., New York, NY, USA), the first PDT for the prevention of migraine. Its development was informed in part by the 2021 AHS Consensus Statement, which assigned a Grade A recommendation to biobehavioral interventions for migraine prevention. According to its FDA labeling, CT-132 is indicated for the preventive treatment of episodic migraine in adults, intended for adjunctive use alongside acute and/or other preventive migraine medications [8]. Given that nearly all individuals with migraine utilize acute treatments, such as nonsteroidal anti-inflammatory drugs (NSAIDs), acetaminophen, triptans, or CGRP inhibitors, CT-132 can be seamlessly incorporated into care plans early in the therapeutic course without necessitating prior pharmacologic failure.

CT-132 also demonstrates a compelling safety profile, with no treatment-related adverse events reported and no contraindications, including for populations often excluded from pharmacologic trials, such as older adults, pregnant individuals, or those with hepatic, renal, or cardiovascular conditions [9]. As a smartphone-delivered, non-pharmacologic intervention, CT-132 presents no drug-drug interactions and requires no dosage adjustments, making it uniquely suited for broad application across diverse clinical settings. This favorable safety and usability profile strongly supports the consideration of CT-132 as a first-line digital therapeutic option in modern migraine care.

A robust digital step therapy protocol can reasonably initiate CT-132 as the first-line intervention for adults diagnosed with episodic migraine, leveraging its safety, tolerability, and real-world usability profile. Treatment progress should be systematically tracked using a multidimensional set of outcomes. From the pivotal clinical trials [9] and collected within the app, these outcomes include migraine frequency metrics such as monthly migraine days (MMD) and monthly headache days (MHD); functional impact measures like the Migraine Disability Assessment Scale (MIDAS) and the Migraine-Specific Quality of Life Questionnaire (MSQ); and patient experience, including the Patient Global Impression of Change (PGI-C). This layered assessment allows both patients and providers to gauge meaningful benefit beyond symptom reduction, capturing quality of life and real-world functioning.

Following the treatment period, patients could be stratified into clinically actionable tiers (Table 2). Those relying solely on acute migraine medications could continue CT-132 if showing benefit, delaying or avoiding the need for preventive drugs. Patients already on preventive therapies, including CGRP inhibitors, could retain CT-132 to optimize multidimensional outcomes and/or reduce pharmacologic burden. For those exhibiting minimal or no benefit, escalation to more intensive therapies may be warranted.

**Table 2:** Digital step therapy pathway for episodic migraine (example) This author-created table outlines a proposed evidence-informed treatment sequence for adults with episodic migraine, integrating CT-132, a prescription digital therapeutic (PDT), with existing pharmacologic regimens. The model supports early initiation of CT-132 alongside acute medications, followed by escalation to combination or advanced therapies based on patient response after 12 weeks. Stratification criteria include clinical metrics of disease frequency (e.g., monthly migraine days (MMDs), monthly headache days (MHDs), functional burden (e.g., Migraine-Specific Quality of Life Questionnaire (MSQ), Migraine Disability Assessment Scale (MIDAS)), patient-experience outcomes (e.g., Patient Global Impression of Change (PGI-C)), and real-world engagement. This pathway aligns with FDA labeling and 2021 and 2024 American Headache Society recommendations for biobehavioral and calcitonin gene-related-peptide (CGRP)-targeted therapies, facilitating personalized, stepwise care grounded in safety, scalability, and outcome tracking. NSAIDs: nonsteroidal anti-inflammatory drugs

Step	Therapeutic approach	Criteria for advancement
1	CT-132 + acute medications (e.g., NSAIDs, acetaminophen, triptans, CGRP inhibitors)	Initiate for all with episodic migraine; assess MMD, MHD, MIDAS, MSQ, PGI-C
2	CT-132 + acute + preventive medications (e.g., CGRP inhibitors)	For those already on preventatives or showing partial improvement
3	Advanced therapies	Minimal/no benefit after 12 weeks or worsening migraine burden

This stratified, outcomes-based approach is both label-concordant and clinically adaptive, aligning with the 2024 AHS position that CGRP-targeting agents no longer require prior treatment failure and that biobehavioral interventions should be deployed earlier. By placing CT-132 at the entry point of migraine prevention pathways, this model reinforces a paradigm shift toward multimodal, personalized care that is proactive rather than reactive, scalable across health systems, and sustainable under value-based reimbursement models.

Conclusion

Digital step therapy represents an essential evolution in payer strategy for managing the growing landscape of FDA-cleared PDTs. By sequencing PDTs based on safety, clinical outcomes, and regulatory indication, payers can ensure that these technologies are not just available but appropriately integrated into standard care. In conditions like migraine, where safe and effective options like CT-132 are now FDA market authorized, digital step therapy can reduce unnecessary use and optimization of pharmacologic agents, increase adherence through robust therapeutic alliance building, and close longstanding treatment gaps to guidelines-based care. Success depends on flexible protocols, equity-minded implementation, and sustained real-world monitoring. If done well, digital step therapy will not only optimize therapeutic value but also empower a more personalized, participatory model of care.
